# Accumulating Evidence for Increased Velocity of Airway Smooth Muscle Shortening in Asthmatic Airway Hyperresponsiveness

**DOI:** 10.1155/2012/156909

**Published:** 2012-12-25

**Authors:** Gijs Ijpma, Oleg Matusovsky, Anne-Marie Lauzon

**Affiliations:** ^1^Meakins-Christie Laboratories, McGill University, 3626 St. Urbain Street, Montreal, QC, Canada H2X 2P2; ^2^Department of Medicine, McGill University, 687 Pine Avenue, Montreal, QC, Canada H3A 1A1; ^3^Department of Biomedical Engineering, McGill University, 3775 University Street, Montreal, QC, Canada H3A 2B4; ^4^Department of Physiology, McGill University, 3655 Promenade Sir William Osler, Montreal, QC, Canada H3G 1Y6

## Abstract

It remains unclear whether airway smooth muscle (ASM) mechanics is altered in asthma. While efforts have originally focussed on contractile force, some evidence points to an increased velocity of shortening. A greater rate of airway renarrowing after a deep inspiration has been reported in asthmatics compared to controls, which could result from a shortening velocity increase. In addition, we have recently shown in rats that increased shortening velocity correlates with increased muscle shortening, without increasing muscle force. Nonetheless, establishing whether or not asthmatic ASM shortens faster than that of normal subjects remains problematic. Endobronchial biopsies provide excellent tissue samples because the patients are well characterized, but the size of the samples allows only cell level experiments. Whole human lungs from transplant programs suffer primarily from poor patient characterization, leading to high variability. ASM from several animal models of asthma has shown increased shortening velocity, but it is unclear whether this is representative of human asthma. Several candidates have been suggested as responsible for increased shortening velocity in asthma, such as alterations in contractile protein expression or changes in the contractile apparatus structure. There is no doubt that more remains to be learned about the role of shortening velocity in asthma.

## 1. Introduction

It has long been known that deep inspirations (DI) in healthy subjects have both bronchoprotective and bronchodilating effects. In asthmatics however these effects are reduced, and in some cases DI exacerbate breathing difficulties. A recent study by Jackson et al. [1] measured the rate at which airway resistance is regained after a DI in methacholine challenged individuals. In asthmatics this rate was significantly higher. Furthermore, while both asthmatics and controls had reduced airway resistance directly after DI, a few minutes later airway resistance in asthma was often increased relative to pre-DI levels, while healthy subjects' airway resistance remained lower. This difference in the rate at which airway resistance is regained is hard to explain with traditional ideas of how airway smooth muscle (ASM) contributes to airway hyperresponsiveness (AHR), that is, increased ASM mass and/or increased ASM force. While non-smooth muscle factors such as parenchymal interdependence may also explain these effects, in the following we argue that an increased ASM velocity of shortening may be responsible for the differential responses between healthy and asthmatic subjects. The rate of shortening of ASM is dependent on the crossbridge cycling rate, the organization of contractile elements within the muscle cells and the internal loading of the ASM cell. The crossbridge cycling rate is high in the early phase of contraction but decreases as the contraction progresses, allowing for the maintenance of force at low energy cost (i.e., latch) [2]. Solway and Fredberg have suggested that smooth muscle with a higher shortening velocity may not benefit as much from the ASM stretches caused by tidal breathing and deep inspirations, as the increased shortening velocity may lead to a faster return to a prestretch length [3]. Furthermore, if an increased shortening velocity is caused by an increase in contractile elements placed in series, the absolute strain and consequently the force per contractile element will be reduced as the strain is distributed over more elements. Consequently, crossbridge cycling will be less affected by strain, resulting in a faster regaining of airway resistance after a DI. 

Our lab set to explore the possibility that an increased shortening velocity in asthma could lead to increased airway constriction under physiological circumstances [4]. In rat trachealis muscle exposed to sinusoidal force oscillations resembling breathing and an occasional DI, we showed that increased shortening velocity correlated with increased total shortening of the muscle and faster recovery after DI. Furthermore, we found that an increase in shortening velocity is as effective, if not more effective, as an equal increase in contractile force. Accordingly, increased airway constriction in asthma does not require increased force generation: an increased shortening velocity would suffice.

Establishing whether shortening velocity changes occur in, and contribute to, asthma is a major challenge. Not only is it difficult to get human ASM, but the interpersonal variability in mechanical response is so large that any but the largest changes will not be noticed [5–7]. Furthermore, it is known that ASM mechanical properties are different for different regions in the lungs [8]. To complicate things further, the measurement of shortening velocity is prone to large variability because of the different procedures used by different labs, the effects of differing methods of force control, the changing velocity with contraction duration and agonist type, and the accuracy of the measurement equipment itself. In this paper we will discuss the varying approaches that have been taken by us and other researchers to assess if and how shortening velocity in asthma is increased and the associated challenges and reservations with each approach.

## 2. Is Shortening Velocity Increased?

Ideally, to confirm an increase in shortening velocity in asthma, ASM tissue from asthmatics and controls are used to assess the shortening velocity directly. However, besides the above-mentioned reservations, shortening velocity varies with age [9] and location in the lung [8] and is likely to be affected by genetic differences and environmental factors. As the procedure for procuring lung biopsies is not harmful to the subject, biopsies can be obtained under controlled conditions with the benefit of having access to detailed medical history and respiratory function parameters and the possibility of collecting data from groups of subjects with similar disease history and background. However, biopsies cannot provide muscle strips, only cells. Bronchial smooth muscle cells from asthmatics have shown an increased rate of shortening and total shortening when exposed to contractile agonists compared to controls [10]. However, the process of obtaining the biopsies and dissociating the cells is likely to have a direct effect on the mechanical response. In addition, the mechanical response of the ASM cell in isolation may not directly correlate with ASM tissues or airways *in situ*. In fact, the total extent of shortening of the unloaded cells is many times greater than is likely to occur *in vivo* and most of this rapid shortening may occur before the cell is fully activated [11]. Consequently, the difference in shortening velocity may be a difference in the rate of activation instead. 

Entire bronchial rings or tissue strips can be obtained from excess tissue from lung resections and pneumonectomies, with a downside of lack of control over subject health status and age. Many studies have been performed on human bronchial rings, but only one has looked at shortening velocity directly [7]. In this study, bronchial rings from nonasthmatic subjects were sensitized overnight with human serum from atopic individuals. While the maximal force remained constant, the shortening velocity and the total amount of shortening increased in sensitized bronchi. This study does not directly prove that shortening velocity is increased in asthma; however, it does suggest a mechanism by which shortening velocity could be increased without changing the force generating capacity of ASM. 

Another approach that has been used to study the pharmacology of bronchoconstriction, and which also yields information about the shortening velocity, is the airway explant [12–15]. In this technique, lungs filled with agarose are sliced transversely and placed under a microscope. The rate of narrowing in response to methacholine has been measured in the Fisher and Lewis rat model of AHR and asthma; a greater rate of shortening was reported in the hyperresponsive Fisher rat [16]. While the lung slice technique has been used to study the dynamics of bronchoconstriction in human airways [17], asthmatic airways have not yet been investigated. One disadvantage of this method of studying ASM mechanics is that it limits the axial continuity of the ASM bundles, resulting instead in patches of ASM cells. The orientation of the muscle bundles may have a profound effect on resistance to airway constriction, and as such the ASM shortening velocity. 

**Figure 1:**
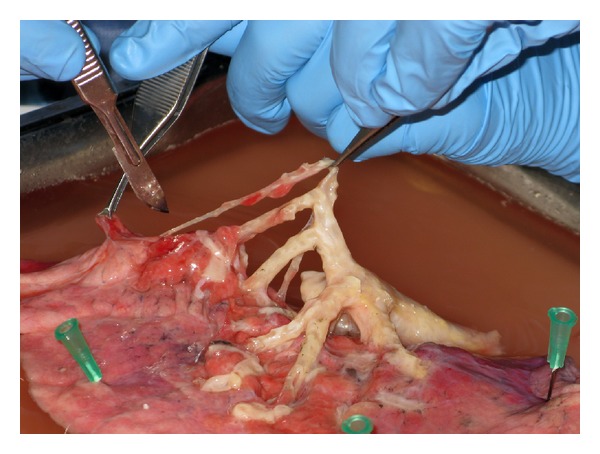
Single lobe dissection of human lung. ASM mechanics from trachea to small bronchi (~1 mm diameter) from both healthy and asthmatic subjects may lead to more conclusive evidence of shortening velocity changes in asthma.

Nowadays, entire donor lungs can be used for research when no suitable transplant recipient can be found (Figure 1). The main benefit of transplant lungs is that repeated investigation of tissues from the same sections in the lung can be performed, reducing at least some of the variability between subjects found in tissues from (partial) lobectomies. Furthermore, as most ASM studies in animals are done on trachealis muscle, the availability of the trachea allows for a more direct comparison with these results. A study by Chin et al. [5] on human trachealis muscle showed a nonsignificant trend of increased shortening velocity in asthmatics. the lack of significant differences seems to be related to large variability in the data, combined with a substantial age difference between the subject groups. As no follow-up studies on respiratory function are possible, it is difficult to assess the quality of the lungs and their position in the healthy-to-severe asthma spectrum. Furthermore, as research centres are generally not in the direct proximity to the tissue source, a considerable delay exists between the harvesting of the lung and the experiments. Studies on isolated airway segments have shown little change in pharmacological response of bronchi stored for more than 2 days [6], but little is known about the effect on ASM of prolonged storage with surrounding tissues (i.e., parenchyma, blood traces, etc.). 

Many studies on animal models of AHR have shown an increase in shortening velocity [18, 19]. However, as the real cause of asthma is as yet unknown, no animal model can be said to mimic asthma, only asthma symptoms. As such, it is irrelevant whether these animal models show increased shortening velocity, what matters is what causes it and does evidence for similar pathways exist in humans. 

## 3. How Is Shortening Velocity Changed?

If increased shortening velocity does play a role in the pathophysiology of asthma, what is responsible for this increase? Molecular level changes could result in faster cross-bridge cycling rates, either through changes in the contractile elements themselves, or through changes in the level of activation. The smooth muscle myosin heavy chain isoform SM-B has been shown to result in a doubling of velocity in motility assays compared to SM-A [20]. The ratio of SM-B/SM-A mRNA is increased in asthmatics [21] but this has yet to be followed by measurements of the protein levels. Furthermore SM-B deficient mice have a decreased rate of airway constriction after methacholine challenge [22]. The crossbridge cycling rate could also be changed independently from differences in myosin isoforms. The gradual change in shortening velocity during a sustained contraction is a strong indicator of variable cycling rates, and most evidence is pointing towards a pivotal role for the myosin light chain phosphorylation level in determining crossbridge cycling rates [2, 23]. Indeed, myosin light chain kinase (MLCK), which phosphorylates the myosin light chain, is increased in asthma at the mRNA level [10, 21] and a variant of the MLCK gene has been associated with severe asthma in African Americans [24]. Furthermore, human bronchi sensitized with serum from allergic asthmatic individuals show increased MLCK levels [25]. An alternative, but not yet tested, theory involves the lengths of the contractile elements within the ASM cells, and its thin filaments in particular. Assuming that the unloaded shortening velocity of an individual contractile element is independent of its length, the total rate of shortening of a cell is determined by the effective number of these contractile elements in series. As ASM is known to allow rapid remodelling, changes in the series to parallel organization of contractile elements are likely to occur, and this is required to explain the constant force at a wide range of lengths in most mathematical models of length adaptation [26–28]. Consequently, a change in the average thin filament length could result in a change in shortening velocity of the muscle [27, 28]. 

Evidence is mounting that the cause for the changes in ASM shortening velocity may have its roots in chronic airway inflammation. Chronic airway inflammation and remodeling underlie the clinical manifestations of asthma. Cultured ASM cells exhibit enhanced contractility and contractile protein expression in response to a number of important cues altered in asthma, including inflammatory mediators [29]. Studies have suggested that airway inflammation is causally related to AHR and these changes could be a result of CD4^+^ T cell activation, which is an important source of inflammatory mediators. An increase in the number of CD4^+^ T cells with a phenotype associated with T-cell activation is found both in bronchoalveolar lavage of asthmatic patients [30] and in the ASM layers of animals with experimental asthma [31]. Lazaar and coworkers [32] originally demonstrated that activated T cells can adhere in vitro to resting ASM cells from nonasthmatic patients. The adhesion was enhanced when ASM cells were primed with proinflammatory cytokines such as tumor necrosis factor-*α* (TNF-*α*). These findings independently confirmed that CD4^+^T cells can interact with ASM not only in vitro [33] but also in vivo [31]. Recently, it has been shown that IL-17A, produced by CD4^+^ T cells, enhanced contractile force generation of human ASM through an IL-17 receptor A [34]. This pathway involves activation of NF-*κ*B (a protein complex that controls the transcription of DNA) and induction of RhoA and ROCK2 expression. ROCK 2 regulates myosin light chain 20 (LC_20_) phosphorylation through inhibition of myosin light chain phosphatase thus promoting the phosphorylated level of LC_20_. These data correlate with studies of Fan and others [19] who found the increasing of MLCK activity and phosphorylation of LC_20_ due to incubation of muscle strips with different inflammatory mediators (including Th2 cytokines) might result in the observed increase of shortening velocity.

## 4. Conclusion

While an increased shortening velocity might not be the only change occurring in asthmatic ASM, it may certainly play a central role in the pathophysiology of asthma. More research is needed to conclusively determine whether, and by how much, shortening velocity is increased in asthma, but the various approaches taken so far provide a very strong indication for an increase as well as likely causes for the increase. Future important findings will probably come from running experiments on tracheal, main bronchial, and intrapulmonary bronchial tissues from whole lungs from transplant programs to assess whether shortening velocity in asthma is changed (Figure 1). Advances in cantilever microfabrication [35], for example, will allow the assessment of loaded cell mechanics by adhering ASM cells directly to a length and a force transducer. This more direct assessment, combined with control experiments on whole ASM tissues, will show whether these cell measurements are indicative of whole-tissue behaviour. This will greatly extend the power of biopsy samples. Furthermore, human lung explants [12–15] could be used for a direct comparison of asthmatic and control airway shortening velocity. All this together will shed light on whether or not the velocity of shortening of ASM is altered in asthma. At this point, some doubt remains especially after the lack of shortening velocity change found recently by Chin et al. [5]. Perhaps future ASM tissue studies can reduce the variability and result in a clearer conclusion on whether shortening velocity is really increased in asthma.
